# Towards a Multifunctional Electrochemical Sensing and Niosome Generation Lab-on-Chip Platform Based on a Plug-and-Play Concept

**DOI:** 10.3390/s16060778

**Published:** 2016-05-28

**Authors:** Adnane Kara, Camille Rouillard, Jessy Mathault, Martin Boisvert, Frédéric Tessier, Hamza Landari, Imene Melki, Myriam Laprise-Pelletier, Elodie Boisselier, Marc-André Fortin, Eric Boilard, Jesse Greener, Amine Miled

**Affiliations:** 1 Electrical and Computer Engineering Deptartment, Faculty of Sciences and Engineering, Université Laval, Québec City, QC G1V 0A6, Canada; adnane.kara.1@ulaval.ca (A.K.); camille.rouillard.1@ulaval.ca (C.R.); jessy.mathault.1@ulaval.ca (J.M.); martin.boisvert.4@ulaval.ca (M.B.); frederic.tessier.1@ulaval.ca (F.T.); hamza.landari.1@ulaval.ca (H.L.); 2 Department of Chemistry, Faculty of Sciences and Engineering, Université Laval, Québec City, QC G1V 0A6, Canada; Jesse.Greener@chm.ulaval.ca; 3 Ophthalmology Department, Faculty of Medecine, Université Laval, Québec City, QC G1V 0A6, Canada; Elodie.Boisselier@fmed.ulaval.ca; 4 Research Center of CHU de Quebec, (Centre Hospitalier Universitaire)-Université Laval, Québec City, QC G1V 0A6, Canada; Imene.Melki@crchudequebec.ulaval.ca (I.M.); Eric.Boilard@crchudequebec.ulaval.ca (E.B.); 5 Centre de Recherche du Centre Hospitalier Universitaire de Québec (CR-CHUQ), Axe Médecine Régénératrice, Québec City, QC G1L 3L5, Canada; myriam.laprise-pelletier.1@ulaval.ca (M.L.-P.); Marc-Andre.Fortin@gmn.ulaval.ca (M.-A.F.); 6 Centre de Recherche sur les Matériaux Avancés (CERMA), Université Laval, Québec City, QC G1V 0A6, Canada; 7 Département de Génie des Mines, de la Métallurgie et des Matériaux, Université Laval, Québec City, QC G1V 0A6, Canada

**Keywords:** lab-on-chip, modular microfluidics, system integration

## Abstract

In this paper, we present a new modular lab on a chip design for multimodal neurotransmitter (NT) sensing and niosome generation based on a plug-and-play concept. This architecture is a first step toward an automated platform for an automated modulation of neurotransmitter concentration to understand and/or treat neurodegenerative diseases. A modular approach has been adopted in order to handle measurement or drug delivery or both measurement and drug delivery simultaneously. The system is composed of three fully independent modules: three-channel peristaltic micropumping system, a three-channel potentiostat and a multi-unit microfluidic system composed of pseudo-Y and cross-shape channels containing a miniature electrode array. The system was wirelessly controlled by a computer interface. The system is compact, with all the microfluidic and sensing components packaged in a 5 cm × 4 cm × 4 cm box. Applied to serotonin, a linear calibration curve down to 0.125 mM, with a limit of detection of 31 μM was collected at unfunctionalized electrodes. Added sensitivity and selectivity was achieved by incorporating functionalized electrodes for dopamine sensing. Electrode functionalization was achieved with gold nanoparticles and using DNA and o-phenylene diamine polymer. The as-configured platform is demonstrated as a central component toward an “intelligent” drug delivery system based on a feedback loop to monitor drug delivery.

## 1. Introduction

The level of LoC complexity is mainly driven by the application. Recently with the emergence of droplet microfluidics, the interest of using LoC became more important. Such interest is due to the fact we are going toward miniaturized devices for micromanipulation and micro-sensing of chemicals and biological fluids. The complexity of LoC can be analyzed from different points of view such as liquid handling, sensing and embedded electronics and also in term of low cost solution and/or easy of use device for medical technology [1]. Huebner at al, in their critical review, presented some aspect of complex of LoC designs related to microdroplets applications within a microfluidic environment [2]. In another review presented by Abgrall and Gué highlighted is the rising complexity of LoC due to the integration of advanced electronics, microfluidics architectures inside the same microdevice [3].

Modern lab-on-chip (LoC) platforms merge microfluidics with one or more functional and/or analytical components, which can include optics, microelectronics, microelectromechanical system (MEMS) and more [4,5,6,7,8]. Integrated microelectronic elements, for example, can handle varied tasks including (i) sensing via voltammetry, amperometry, capacitance, impedance spectroscopy; (ii) stimulation and actuation via applied current voltage, dielectrophoresis, field flow fractionation and electrowetting; (iii) data-acquisition and (iv) data treatment via digital signal processing elements [9,10,11,12], Some applications include gluco-sensors [13], breath alcohol measurements [14], integrated fuel cells, brain electrochemical monitor [15], droplet manipulation and sorting [16], and even imaging [17]. However, new approaches that integrate higher-level functionality are required to further propel LoCs platforms toward new applications. At the same time, devices must not become too complex in order that they can be operated by a wide range of users. Component modularity is one way forward, which can allow users to reconfigure their system as the need arises. The concept of plug-and-play in personal computers serves as a model for future LoC development.

As the complexity of LoC is increasing constantly, users are facing more challenges in handling such systems. The concept of plug-and-play is well know with computers. It consists of connecting any accessory to the central unit and it will be ready for use immediately. Other researchers proposed a microfluidic plug-and-play system. However, it was mainly limited to the microfluidic part. Indeed LoC is an integration of electronics with microfluidics/MEMS. Then, only microfluidics/MEMS plug-and-play concept is not enough to achieve a high versatility, as it is the case with computers nowadays where each module is used for a specific purpose. However, users do not really need to use all of them at the same time. Based on this concept, we proposed a LoC architecture where the liquid control, sensing and communication modules are fully independent depending on the required setup. LoC can be divided mainly into two main categories of modules, which are actuation and sensing. We will focus in this paper on electrochemistry as sensing system.

While some advances have been made in the microfluidic plug-and-play concept [18,19] and reconfigurable analytical probe [20], system-level reconfigurability of complex LoC platforms has yet to be demonstrated. Indeed, LoC is an integration of electronics with microfluidics/MEMS [21,22,23,24]. But, only microfluidics/MEMS plug-and-play concept is not enough to achieve a high versatility, as it is the case with computers nowadays where each module is used for a specific purpose. For example, in the case of computers, users do not really need to use all computer performances (Graphical processor, computing processors, accessories…) at the same time but depending on the application module performances will be optimized. Based on this concept, we proposed a LoC architecture where the liquid control, sensing and communication modules are fully independent depending on the required setup. LoC can be divided mainly into two main categories of modules, which are actuation and sensing. We will focus in this paper on electrochemistry as sensing system and noisome generation.

Renaudot *et al.*, presented a concept of a microfluidic platform which is based on reconfigurable and addressable electrode array with a surrounding medium of liquid-phase paraffin. Authors claim that their process is resettable and reversible by melting the paraffin [22]. Banaerjee *et al.*, developed a reconfigurable virtual electrowetting channels by reconfiguring the setup of in-channel electrodes to create virtual channels based on the electrowetting concept for rapid prototyping platform [22]. Other authors addressed other issues related to the reconfigurability of lab-on-chip and/or microfluidic such as reversible sealing or reconfigurable capillary connection [23,24]. All previously cited example, addressed the reconfigurability aspect from the microfluidic aspect. Lab-on-Chip is a multi-technology platform where electronics/microelectronics is connected to microfluidics/MEMS, pumps and valves and vice versa. Then, in order to make the LoC application independent as much as possible, it is important that the LoC become reconfigurable from system level aspect including microfluidics, electronics, flow actuation and also the software part. This aspect is presented in this manuscript and we developed a LoC which is highly reconfigurable from the microfluidic aspect (in-channel electrodes), flow actuation (pumps) and sensing part (electrochemical detection). Furthermore, the concept presented in this paper is based on the high modularity. Then most of modules of the LoC can work independently from each other’s or connected together to form one complete system through the software interface.

Then our goal in this first version of the Plug-and-Play (PnP) LoC work was to build the system and test each functional element individually. Then we reconfigure the device to do each one of tests.

Electrochemistry in LoC platform is becoming more attractive as it offers a powerful portable tool for different type of analysis such as gluco-sensors [13], determination of breath alcohol [25], integrated fuel cells or brain electrochemical activity [15] and even imaging [17]. Our interest in this project is related to the detection of electrochemical activity of NT molcules in brain.

In much of the brain, NTs and neurons are surrounded by the cerebrospinal fluid, which is highly ionic, composed mainly of water, sodium and chloride ions [26,27]. For example, dopamine, produced by neurons from the substantia nigra and ventral tegmental area in the midbrain, is involved in the control of our movements. However, an imbalance in the concentration of these NTs can lead to neurodegenerative diseases such as Parkinson’s disease, Alzheimer’s disease and schizophrenia. Current treatments for these diseases consists mainly of medication given all day long. However, changes in NT concentration during the day and night are frequent, so it is important to sense the most accurate concentration of NT to deliver the adjusted quantity of drug with a local drug delivery system. NTs does not exist only in brain but also in many other area and organs in body [28]. More particularly, serotonin is one of the NT which exists in blood and that is related to several diseases such as coronary atherosclerosis [29], in early infantile autism [30]. Blood platelets represent the main pool of circulating serotonin. Serotonin, stored in platelet, can be released in blood circulation following a platelet activation [31]. Studies showed that in rheumatic diseases, such as rheumatoid arthritis and systemic lupus erythematosus, platelets are activated and release their broad arsenal of mediators, including serotonin [31]. Hence, platelet-derived serotonin impact the permeability of the vasculature during inflammation, and mouse models are used to monitor serotonin functions in diseases [32].

Therefore “intelligent” dosing, a central concept in personalized medicine, should incorporate sensing of NT and other biomarkers so that the right levels can be prescribed [33]. The modular LoC platform demonstrated here is the core of an intelligent NT dosing system. Liquid handling allows real-time generation of niosomes of different dimensions for drug delivery and electrochemical methods such as CV and chronoamperometry are used to detect NTs concentrations. Serotonin was detected on bare, functionalized electrodes, whereas a functionalization was required for detection of dopamine.

**Figure 1:**
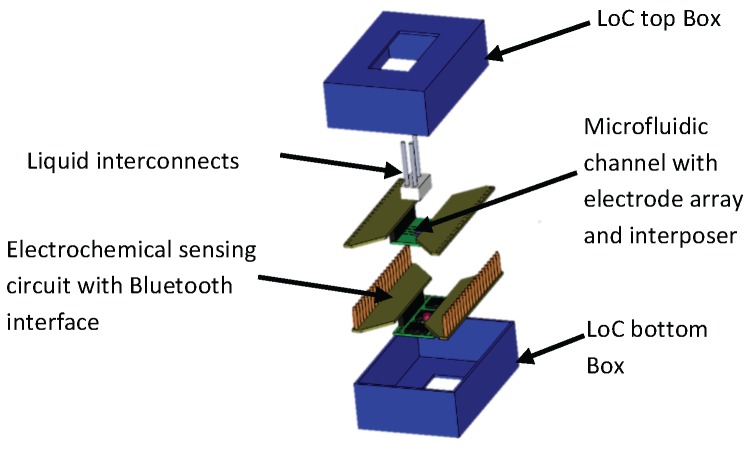
Proposed architecture of the PnP LoC design including microfluidics, interposers, electronics and packaging.

Based on the PnP concept, we demonstrate a modular LoC system, which includes a three-channel liquid handling system, a microfluidic compartment with an embedded electrode array connected to a multiplexer as shown at Figure 1. A three-channel potentiostat is capable of conducting cyclic voltammetry or chronoamperometry measurements. A computer running custom software provided control over all elements via USB or wireless connection. The elements of the system were specifically adapted for NT modulation/detection in laboratory animals. These include: on-chip niosome generation for drug-delivery [34,35], and separate detection channels for dopamine and serotonin. Measurements of NTs were enhanced by functionalized electrodes, which were validated in a cerebrospinal fluid model (PBS buffer) and in real human blood plasma.

## 2. General System Description

**Figure 2:**
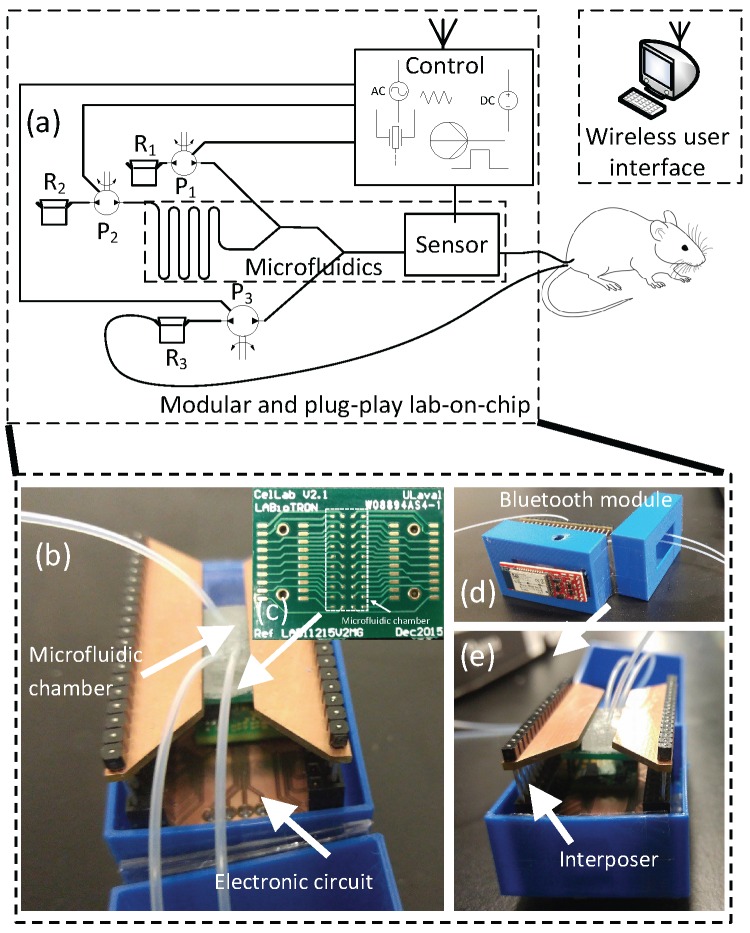
(**a**) LoC modular and PnP schematic of functional each module (microfluidic/sensor, microcontroller and fluid handling). The microcontroller part consists of Arduino nano implemented with the potentiostats circuits and the micropumping module. Interface with computer is wireless or USB; (**b**) Self-contained LoC with outer dimensions 5 cm × 4 cm × 4 cm (defined by blue box). LoC PnP internal architecture showing microfluidic chamber, potentiostat and other electronic circuitry; (**c**) Electrochemical reaction area inside the microfluidic chamber; (**d**) Bluetooth wireless communication and (**e**) interposers for connection between different modules.

In this work, three separate modules were developed which could work independently or together as shown in Figure 2. The microfluidic module consisted of different channel geometries in polydimethylsiloxane (PDMS) with a sealing layer provided by a printed circuit board (PCB) containing a 4 × 20 gold electrode array which has been used previously for electrochemical imaging [17].

**Figure 3:**
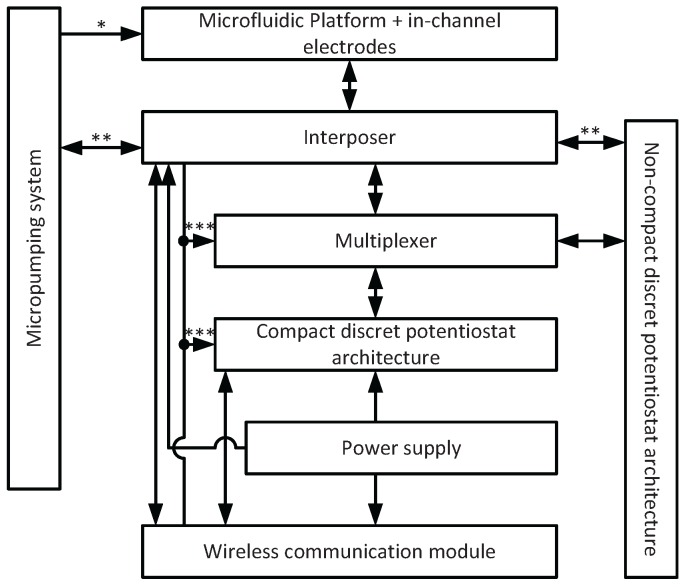
Schematic illustration of the proposed LoC architecture including the connection of the interposer with other electronics modules. * refers to the tubing between the microfluidic platform and the micropumping system. ** refers to optional connections; these optional connections are related to power supply and wireless communication. *** refers to optional power supply distribution through the interposer.

As shown at Figure 3 the interposer connects each electrode to the second microelectronics module. This consisted of a multiplexing system for selecting working, counter and reference electrodes throughout the entire electrode array and a custom-designed 3-channel potentiostat. The latter was used for sensing different target NTs using cyclic voltammetry or chronoamperometry. Interposer has been added several optional connections to distribute the power supply from one main module to all the rest of the architecture. Also an optional connection has been added to handle wireless communication through the interposer. However, for higher modularity, we kept these connections optional while the micropumping system can handle its own wireless communication.

**Figure 4:**
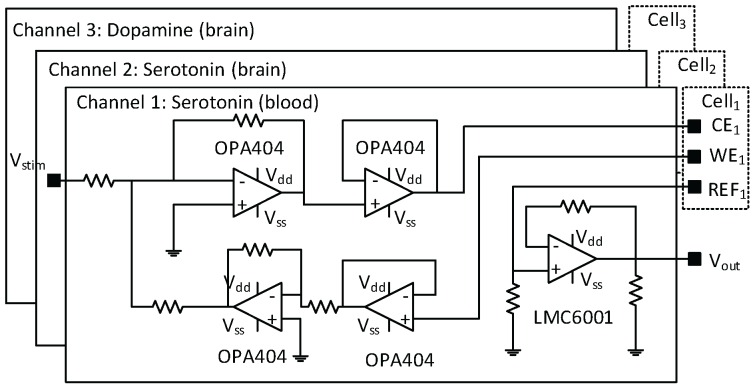
A three-channel potentiostat implanted on PCB. Each channel is designed to detect one molecule (serotonine, dopamine 1 or dopamine 2). The input signal (Vstim) is shared between all potentiostats. Vout is the measured current.

Though the boards could handle frequencies up to 165 kHz, measurements were limited to 25 Hz because modulation of liquid environment was relatively slow at flow rates used here. Applied voltages ranged between −1.2 V and 1.2 V. Static power consumption for each potentiostat channel shown at Figure 4 of the LoC was 95 mW, while the static power consumption of all other components for electrochemical sensing was 165 mW in the stand-by mode. Such power consumption is acceptable for remote and/or PnP LoC. For both the potentiostat circuit and the multiplexing system a control electronic board based on field-programmable gate array (FPGA) and microcontroller board (Arduino) were designed with software interface through (LabVIEW, National Instruments, Austin, TX, USA).

Finally, the liquid handling module was composed of three peristaltic micropumps, each one was able to be set on infusion or withdraw mode, with a complete system stop for when pumping reservoirs are empty. Peristaltic micropumps has been chosen because they are much easier to clean in biological experiments by only replacing the tubing. Two set of micropumps have been tested (RB-Ada-91, Robotshop, Canada and 3200243, Dolomite, UK). The first is mainly applicable for high flow rate applications. For higher accuracy, the second was used. Proposed LoC was able to handle flow rates between 0.2 mL/min and 0.45 mL/min. The flow rate of peristaltic micropumps was linear versus motor rotation.

In addition to hardware interfacing, software modularity enabled seamless operation. This controlled all potentiostat experimental parameters (scan rate, voltage sweep range, and electrode positions in the in-channel array), data transmission and all fluid handling aspects. Table 1 shows the principal features of the modular LoC developed here. All modules were designed to be interchangeable and reconfigurable. In this work we demonstrate the system for niosome generation, dopamine detection for brain applications and serotonin sensing in blood. However, it is worth noting that the system covers more features than targeted applications presented in this paper.

## 3. Experimental Setup

### 3.1. System Configuration

**Figure 5:**
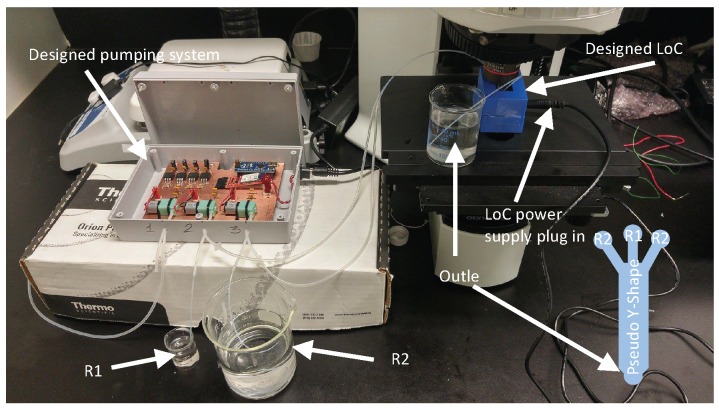
Experimental setup of the proposed LoC where reservoir (2) contains a mixture of sorbitan monolaurate, cholesterol and dicetyl phosphate dissolved in isopropyl alcohol (IPA) and (3) PBS.

Figure 5 shows experimental setup of the designed LoC.

**Figure 6:**
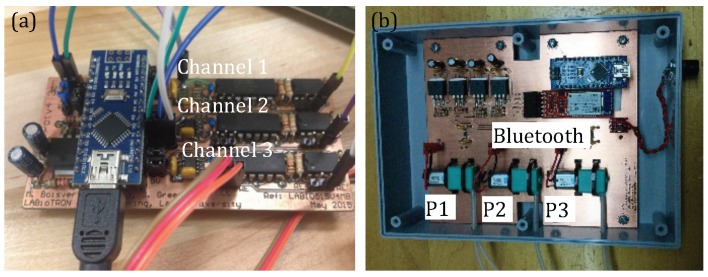
(**a**) 3-channel potentiostat where each channel is dedicated to one measurement (serotonin, dopamine 1 and dopamine 2); (**b**) The micropumping system of this platform consists of 3 channels (P1, P2 and P3) controlled by peristaltic micropumps from dolomite. An embedded microcontroller (Arduino Uno) handle the control part of all electronic system.

For practical reason, two potentiostats were been designed: (i) Compact version, which was implemented directly in the LoC, and (ii) another version shown at Figure 6 which was used during this test. (i) and (ii) are the same but (ii) is easier to manipulate as the compact version is assembled in a compact way.

**Figure 7:**
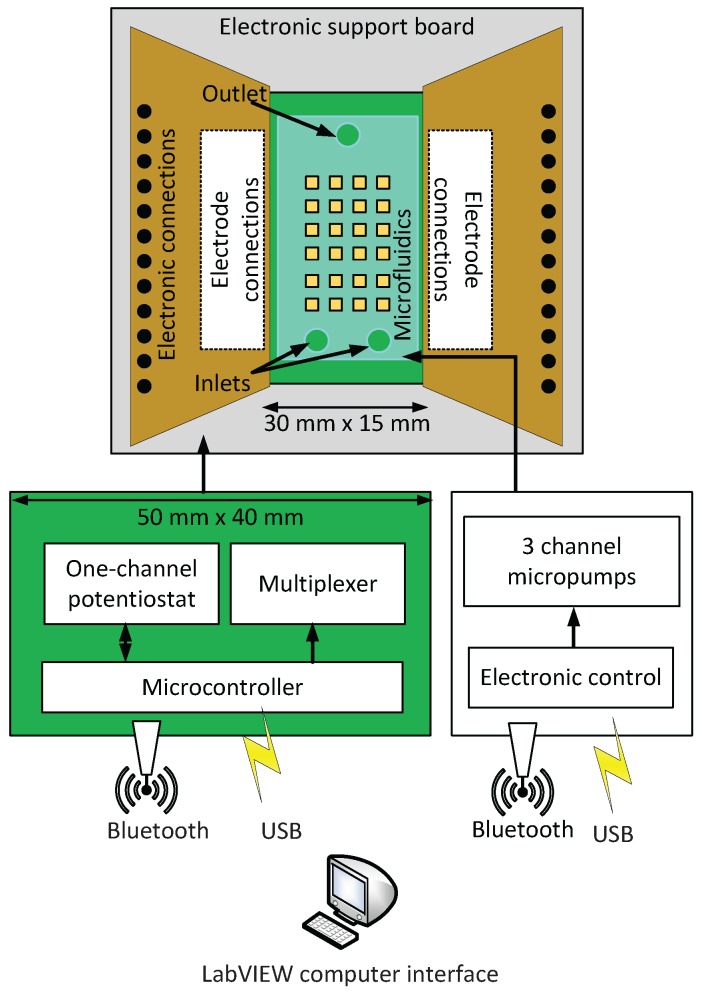
Multifunctional LoC diagram. All modules can interface with each other’s through a LabVIEW interface. They can handle both wireless and USB communications depending on the application.

The PnP concept of the proposed LoC is shown at Figure 7. The microfluidic chamber with embedded electrodes can be removed and replaced independently from the other modules. This was achieved through the interposer. The wireless communication module was placed outside the box in order to avoid interferences. Furthermore, it is removable and can be replaced by USB connection. The electronic circuit embeds the multi-channel potentiostat and the multiplexing system.

**Figure 8:**
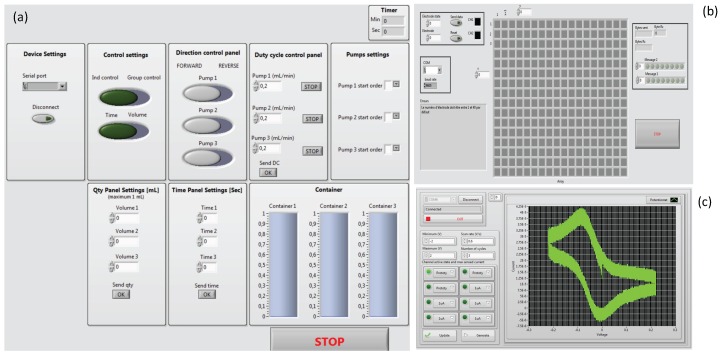
Designed LabVIEW interface (**a**) micro-pump control (**b**) electrode array control and (**c**) 3-channel potentiostat interface.

All modules of the LoC were independently controlled by the LabVIEW interface as shown at Figure 8. The software is extremely automated so that if any reservoir is going to be empty its corresponding micropumps is automatically stopped. The potentiostat interface monitors each sensing channel, which could be set with different sensitivities. The electrode array control interface was able to connect each channel of the potentiostat to any electrode in the microfluidic chamber or other electrode connected externally to the LoC.

### 3.2. Niosome Generation System

Preparation of precursors: Sorbitan monolaurate (Span 20) was purchased from Alfa Aesar (Ward Hill, MA, USA), dicetyl phosphate (DCP; C32H67O4P; Molecular Weight 546.85) was ordered from MPBiomedicals (Solon, OH, USA), cholesterol was purchased from Avanti Polar Lipids (Alabaster, AL, USA). Phosphate buffered saline (1x PBS) was from Sigma-Aldrich, as well as chloroform and isopropyl alcohol (IPA). In brief, span, cholesterol, and DCP were dissolved in chloroform and prepared in a molar ratio of 47.5:47.5:5.0, respectively, at a total of 10 mg/mL in a glass scintillation vial. The chloroform solvent was evaporated under nitrogen gas to form a dry surfactant film on the bottom of the scintillation vial. The vial was then placed into a vacuum desiccator for at least 18 h to ensure complete solvent removal. The dried surfactant mixture was resolubilized in IPA at 5 mmol/L concentration, followed by filtration (0.2 microns) and centrifugation (3000 x g, 30 min). PBS at pH 7.4 was used as the hydration buffer for the niosome self-assembly in the microfluidic devices.

**Figure 9:**
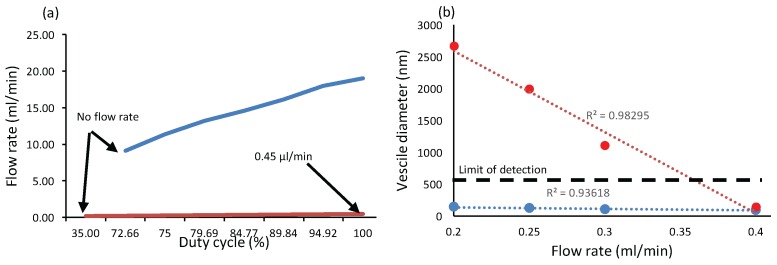
(**a**) Flow rate of two designed micropumping systems for proposed LoC system. Red and blue curves correspond to the low and high flow rate pumping system and (**b**) Vesicle diameter with different flow rate. Red curve corresponds to the cross shape PDMS channel while the blue colour is the pseudo-Y shape channel. In the case of the pseudo-Y channel vesicle diameter varies between 91.8 nm and 141.8 nm, which is considerably lower than the case of cross-shape.

Measurements: Two micro-pumping systems were tested and reported at Figure 9a: (i) RB-Ada-91, Robotshop, Canada (blue) and (ii) part # 3200243, Dolomite, UK (red). RB-Ada-91 has not been used for our applications as the minimum flow rate of this system was 11.34 mL/min which was too high. Also Figure 9b reports characterized vesicles size from the outlet of the microfluidic system. However, obtained results related to vesicles larger than 500 nm were not reliable as the limit of detection of used algorithm with DLS was 500 nm.

### 3.3. Fabrication of Functionalized Electrodes for Enhanced Sensitivity and Selectivity of Dopamine Detection

**Figure 10:**
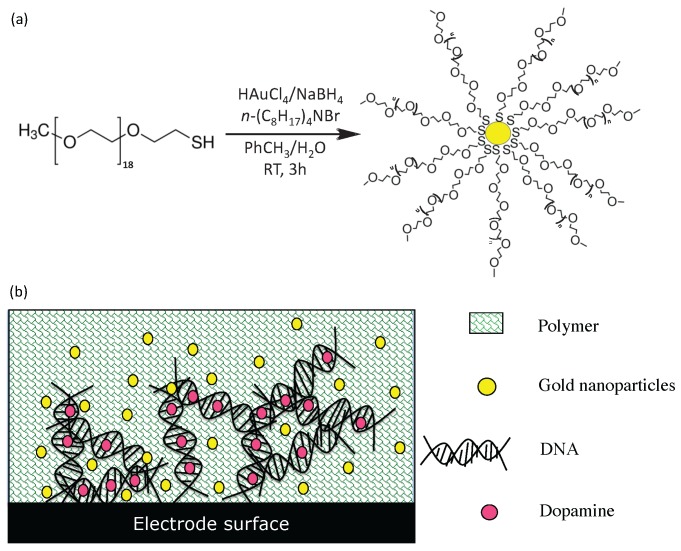
(**a**) Gold nanoparticles synthesis using modified Brust method with polyethylene glycol ligand and (**b**) electrode surface functionalization principle using gold nanoparticles.

Electrode functionalization using gold nanoparticles was achieved using DNA, O-PD polymers and gold nanoparticles. First, gold nanoparticles with 7 ± 3 nm inner diameter (by transmission electron microscopy, data not shown) were synthesized using modified Brust method [36] (Figure 10).

Briefly, thiolated ligands were placed in contact with chloroauric acid before addition of sodium borohydride to achieve the reduction of gold (Au${}^{3+}$ to Au${}^{0}$). Then, two purification steps were performed: an extraction with dichloromethane to remove salts and a precipitation with ethanol/diethyl ether to remove ligand in excess. The thiolated ligands used to stabilize the gold nanoparticles are composed by polyethylene glycol groups (PEG). These PEG ligands are also biocompatible and make the gold nanoparticles soluble and stable in water. Chemically reduced gold nanoparticles have several advantages in electrochemistry. Indeed (i) they are highly conductive and biocompatible (ii) they allow a transfer of kinetic faster electrons and (iii) they increase the electro-active surface of the electrode.

In order to improve the sensitivity of the system to dopamine, a pyrolytic graphite carbon electrode was functionalized. Initially, this electrode has undergone a pre-treatment in a solution of lithium perchlorate (0.1 M) and sodium carbonate (0.1 M) using cyclic voltammetry (−0.3 V to 1.2 V, scan rate 10 V/s, 5 cycles). This electrode was then functionalized with DNA, o-phenylene diamine polymer and gold nanoparticles (ESI) [37]. This functionnalization is based on the fact that DNA can be linked with high affinity to dopamine by electrostatic and hydrogen bonds. Gold nanoparticles in the solution increased the sensitivity of the electrode and enhance the electrochemical response, as previously reported [38,39]. After the addition of DNA, dopamine and gold nanoparticles to the solution surrounding the electrode, the monomer o-phenylene diamine was added and electrochemically polymerized. This immobilized all the compounds onto the electrode surface, conferring enhanced durability and stability to the functionalization layer. A washing step was then carried out to remove only the dopamine, which was used to form the template. Dopamine binding sites were, therefore, unchanged and will be able to bind and detect dopamine during the electrochemical assay.

## 4. Results

### 4.1. Niosome Generation Module

Niosomes are synthetic membrane vesicles formed by self-assembly of nonionic surfactants, often in a mixture with cholesterol and dicetyl phosphates. Niosomes are commonly used as carriers of treatment agents for pharmaceutical and cosmetic applications or contrast agents for clinical imaging applications; they are considered more stable than liposomes [40]. Recently, the possibility to produce niosomes using microfluidic hydrodynamic focusing, has been demonstrated [40]. During niosome generation, all the sensing modules (potentiostat) were disabled and only micropumps connected to microchannel were enabled. Sensing modules has been disabled because it was not useful for niosome generation in our experimental protocol. Indeed niosome diameter measurement has been achieved off-line by dynamic light scattering (DLS) and to avoid any interferences [18].

Figure 2 shows the three reservoirs containing (1) a mixture of sorbitan monolaurate, cholesterol and dicetyl phosphate dissolved in isopropyl alcohol (IPA), whereas reservoirs 2 and 3 contain PBS. All reservoirs were connected to a cross-shaped and pseudo-y shaped microfluidic channels for noisome generations. The surfactant mixture (QS) was injected in the central channel at a fixed flow (0.2 mL/min), whereas the two lateral flows (QB: PBS) were varied from 0.2 to 0.4 mL/min. Therefore, the flow rate ratio (FRR = 2QB/QS)), defined as the ratio of the PBS volumetric flow rate (QB) to the IPA volumetric flow rate (QS), was varied from 2 to 4. The hydrodynamic diameters of the niosome solutions were measured by DLS with a Nano S Zetasizer system (Malvern Instruments, Worcestershire, UK.) using a laser (He-Ne) wavelength of 633 nm and a scattering angle of 173 ${}^{\circ }$. The temperature measurement was fixed at 25 ${}^{\circ }$C. The viscosity and the refractive index of water were set to 0.8872 cp and 1.33, respectively.

**Figure 11:**
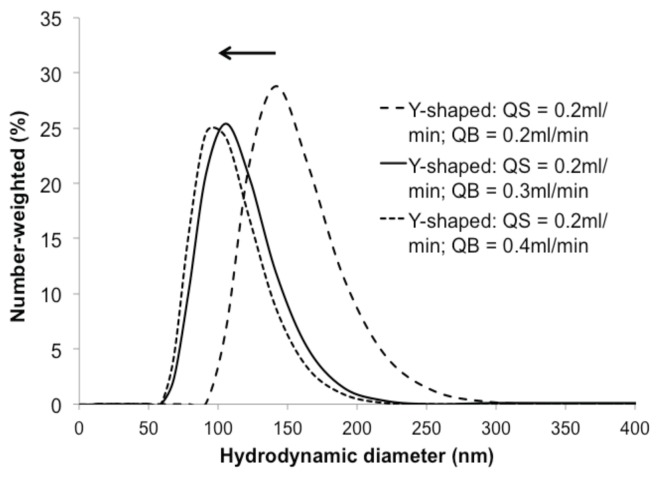
Obtained vesicle diameters with different flow rates and microchannel architectures. In the case of the pseudo-Y channel vesicle diameter varies between 91.8 nm and 141.8 nm, which is considerably lower than the case of cross-shape.

The DLS hydrodynamic profiles in diameter of niosomes produced in the pseudo-Y shaped channel showed the presence of colloids of 50–250 nm diameter. A clear tendency toward smaller niosome sizes was observed for higher FRRs (QS = 0.2 mL/min; QB = 0.4 mL/min; FRR = 4). These results are in agreement with previous studies on niosome synthesis with microfluidic devices [40]. Much coarser and irregular size diameters were found for syntheses produced with the cross-shaped duct (results not shown). Niosomes produced by the pseudo-Y shape duct had more stable and reproducible diameters (maximum from 141 nm down to 91.8 nm, as shown at Figure 11) These results indicate the possibility to achieve a better size control of the niosomes, by increasing FRR. This could be achieved either by decreasing the size of the ducts (decreasing the flow rate), or by increasing QB (thus leading to the production of larger volumes). Coupled with feedback from electrochemical NT detection and microfluidic filters to make the obtained solution biocompatible, this module could modulate drug delivery parameters for personalized medicine.

### 4.2. Electrochemical Detection of Serotonin

**Figure 12:**
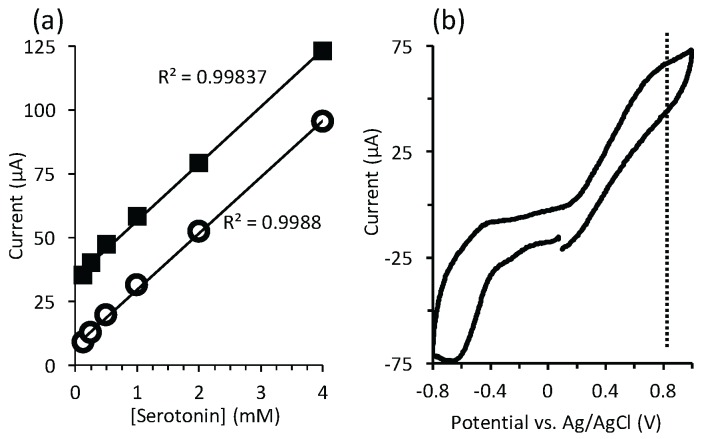
(**a**) Linearity of detected current in the case of serotonin with concentrations down to 0.125 mM using cyclic voltammetry (solid squares) with scan rate 150 mV/s and chronoamperometry (open circles); error bars in (**a**) were smaller than the data points; (**b**) CV curve of 8 mM of serotonin in human blood plasma. Scan rate : 150 mV/s. The vertical line (dashed) shows the position of the peak that was analysed in (**a**).

Next we demonstrated the ability to monitor serotonin in different relevant liquid phases. For this purpose, the PDMS layer was removed, exposing the electrode array so that an Ag/AgCl reference electrode could be inserted for accurate characterization of redox potentials. After that robust gold pseudo-reference electrodes were used as shown previously [17]. Using CV, an easily resolvable, concentration-dependent oxidation peak ca. 0.8 V for serotonin in PBS. We could also see a concentration-dependent equilibrium current using chronoamperometry. By analyzing the peak intensity from the CV and chronoamperometry measurements, after background subtraction using a 0 mM sample, we observed a linear reduction in current with concentration down to 125 μM (Figure 12a). Due to a more facile background subtraction in the case of chronoamperometry, the y-intercept of the linear regression was closer to 0 A than for CV. In both cases, the R${}^{2}$ value of the linear regression was greater than 0.998. The current continued to decrease monotonically with the concentration down to a limit of detection at 30 μM, but linearity was lost. Using CV, an easily resolvable, concentration-dependent oxidation peak has been observed at 0.8 V for serotonin in PBS. We could also see a concentration-dependent equilibrium current using chronoamperometry. By analyzing the peak intensity from the CV and chronoamperometry measurements, after background subtraction using a 0 mM sample, we observed a linear reduction in current with concentration down to 125 mM (Figure 12a).

Next, a serotonin solution in human blood plasma was injected into the LoC system to test its viability for real clinical conditions. Figure 12b shows obtained results, in which the signature oxidation peak was observed at 0.8 V. We noted some instability in the signal over multiple CV cycles, which was not observed for the measurements in PBS. This is likely the result of electrode fouling due to non-specific adsorption from components in the blood plasma.

### 4.3. Electrochemical Detection of Dopamine Module

As dopamine could not be selectively detected at the bare electrode, surface electrode functionalization was undertaken. The use of gold nanoparticles with DNA and o-phenylene diamine polymers at their surface was found to improve the sensitivity and selectivity of dopamine [37]. A new method of functionalization using small gold nanoparticles (7 ± 3 nm) was used, as detailed in the ESI. In addition to targeted improvements to dopamine sensing, modified electrodes based on conductive gold nanoparticles have the advantage of high conductivity and good electron transfer kinetics, biocompatibility and increasing electrode surface area.

**Figure 13:**
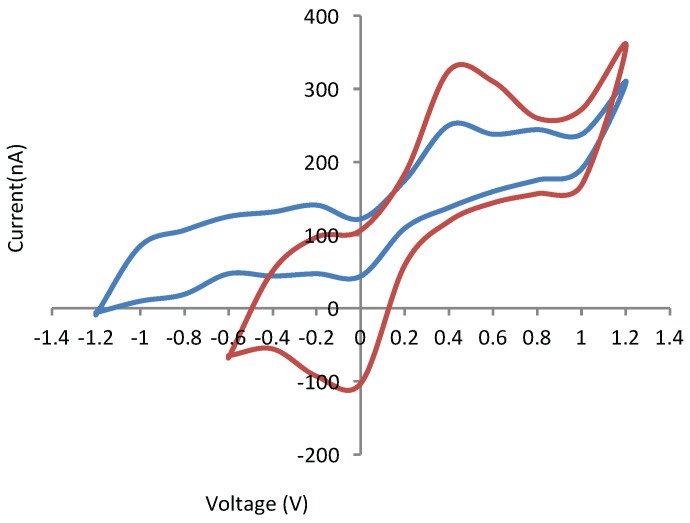
Detection of 7 mM dopamine with non-functionalized (blue) and functionalized (red) carbon electrode.

A dopamine solution in concentrations ranging from 4 mM to 8 mM was prepared to validate the ability to selectively sense this NT. The solution was prepared in a PBS solution to mimic cerebrospinal fluid. As seen in Figure 13, a well-defined peak (0.4 V, 325 nA) was resolved, in comparison to a noisy, unresolved peak on a non-functionalized electrode. The signal was stable over at least 5 cycles. In addition, selectivity against a co-dissolved glutamate NT (7 mM) was also conferred (ESI).

## 5. Conclusions

In this work, we demonstrate a new modular concept in LoC devices, which combines microfluidic flow channels of different geometries with embedded electrode arrays, control electronics for electrochemical detection and a liquid handling system, all controlled by a central software system. We configured the system to form the core of an intelligent dosing system, which will simultaneously dose noisome drug carriers, while measuring dopamine and serotonin in cerebrospinal fluid and blood. We were also able to detect dopamine in a non-homogeneous solution with no background signal from other NT. Such unique system will boost the biomedical research in order to understand how NTs behave in blood and brain and what is their relation in a range of diseases, such as Alzheimer, Parkinson and in rheumatic diseases. Further improvements of the system consist of parallel measurements at different positions on the electrode array with specific functionalizing for different NTs and biomolecules. As the system itself is flexible and reconfigurable, it can open the way for a wide range of new and unrelated applications.

## Figures and Tables

**Table 1:** Modular LoC versus used application specification.

Application Specifications			
	Serotonin	Dopamine	Niosome
Liquid actuation	Pumping system: withdraw/infusion	Pumping system: withdraw/infusion	Pumping system: infusion
Sensing	Electrochemical	Electrochemical	No sensing
Data acquisition	Yes	Yes	No
**LoC specifications**			
Pumping	Infusion/Withdraw		
Sensing	Electrochemical		
# channels	3 independent potentiostat channels		
# sensing site	40 sites. Each site corresponds to one electrode		
Voltage range	−1.5 V to 1.5 V (stimulation)		
Maximum Frequency	165 kHz (stimulation)		
Wave shape	Sine, triangular or square (stimulation)		
Microfluidics	Pseudo-Y-shape chamber, consumable		
Interface	USB or Wireless		
Software	LabVIEW		

**Figure A1:**
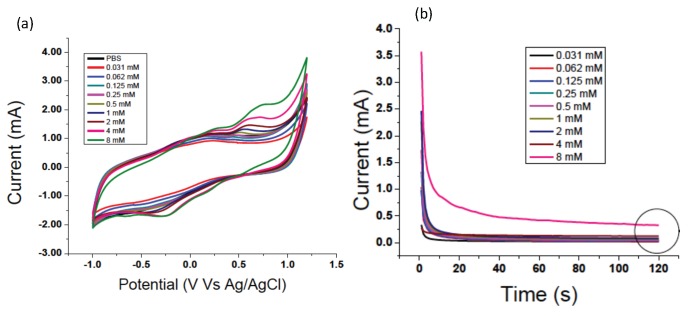
(**a**) CV and (**b**) chronoamperometry curves of serotonin with different concentrations in PBS. The circle in (**b**) shows the times at which the current measurements were acquired for Figure 12a in the main paper. In all cases, electrode surface was 700 μm × 700 μm.

**Figure B1:**
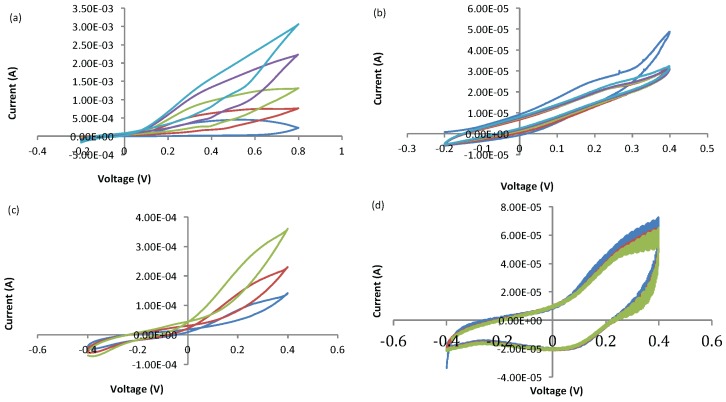
Measurement of gold electrode functionalization by CV: with (**a**) DNA, O-PD and AuNPs; (**b**) thick gold layer; (**c**) carbon nano tubes and (**d**) CNT embedded in chitosan. Each CV colours represent a different cycle . Changes to CV curves in (**a**,**d**) indicate some instability. Scan rates for all measurements were 150 mV/s.

## Appendix A. Serotonin Detection

Figure A1 shows non-background-subtracted CV and chronoamperometry results.

## Appendix B. Gold-Based Electrode Experiments

Bare gold electrodes were also used. During the functionalization, they were oxidized rapidly and could not be used for further testing (Figure B1). One of the major problems with the gold electrodes is that they oxidise at a low potential (about 0.5 V), making the DNA deposition, polymer nanoparticles difficult, since it must be made up to a potential of 0.8 V. In addition, used PCB–based electrodes were made with several different metals, such as nickel, graphite and gold which did not form a uniform surface. Several electrodes post-processing were undertaken to improve sensitivity. Then, nickel was deposed on the electrode followed by a thick layer of gold, both by electro-deposition. The reported electrochemical functionalization was then applied again. We did not observe any peak for dopamine. Other experiments were also achieved with carbon-nanotubes (CNT) on a bare electrode and also on a modified electrode with chitosan. In the latter, they are arranged vertically, thereby increasing the accessibility of their ends, in contrast to a horizontal disposition.

The bare gold electrode array was also tested with dopamine only and PBS. Obtained results have been reported in Table B1. We can clearly see that electrodes were very stable. However as mentioned before the main problem with this electrode is the selectivity.

**Table B1:** Electrode Array characterization.

	$E_{a}$ (V)	$E_{b}$ (V)	$E_{a}-E_{b}$ (V)	$I_{a}$ (μA)	$I_{b}$ (μA)
Electrode 1	−0.07	0.16	0.23	−1.50	2.09
Electrode 2	−0.07	0.16	0.23	−1.16	1.70
Electrode 3	−0.07	0.14	0.21	−1.47	2.05
Electrode 4	−0.07	0.14	0.21	−1.26	1.78
Electrode 5	−0.08	0.15	0.23	−1.90	2.30
Electrode 6	−0.07	0.13	0.20	−1.35	1.80
Average	−0.07	0.15	0.22	−1.44	1.95
Standard deviation	0.003	0.011	0.012	0.259	0.231
